# Transplantation of PDGF-AA-Overexpressing Oligodendrocyte Precursor Cells Promotes Recovery in Rat Following Spinal Cord Injury

**DOI:** 10.3389/fncel.2017.00079

**Published:** 2017-03-21

**Authors:** Zong-Feng Yao, Ying Wang, Yu-Hong Lin, Yan Wu, An-You Zhu, Rui Wang, Lin Shen, Jin Xi, Qi Qi, Zhi-Quan Jiang, He-Zuo Lü, Jian-Guo Hu

**Affiliations:** ^1^Department of Clinical Laboratory, The First Affiliated Hospital of Bengbu Medical CollegeBengbu, China; ^2^Anhui Key Laboratory of Tissue Transplantation, Bengbu Medical CollegeBengbu, China

**Keywords:** platelet-derived growth factor-AA, oligodendrocyte progenitor cells, transplantation, spinal cord injury, functional recovery

## Abstract

Our previous study showed that Schwann cells (SCs) promote survival, proliferation and migration of co-transplanted oligodendrocyte progenitor cells (OPCs) and neurological recovery in rats with spinal cord injury (SCI). A subsequent *in vitro* study confirmed that SCs modulated OPC proliferation and migration by secreting platelet-derived growth factor (PDGF)-AA and fibroblast growth factor-2 (FGF)-2. We also found that PDGF-AA stimulated OPC proliferation and their differentiation into oligodendrocytes (OLs) at later stages. We therefore speculated that PDGF-AA administration can exert the same effect as SC co-transplantation in SCI repair. To test this hypothesis, in this study we investigated the effect of transplanting PDGF-AA-overexpressing OPCs in a rat model of SCI. We found that PDGF-AA overexpression in OPCs promoted their survival, proliferation, and migration and differentiation into OLs *in vivo*. OPCs overexpressing PDGF-AA were also associated with increased myelination and tissue repair after SCI, leading to the recovery of neurological function. These results indicate that PDGF-AA-overexpressing OPCs may be an effective treatment for SCI.

## Introduction

Spinal cord injury (SCI) leads to cell loss and the destruction of descending motor and ascending sensory tracts, resulting in permanent neurological deficits with limited prospects for spontaneous recovery (Quencer and Bunge, 1996). A number of SCI treatment strategies to promote endogenous repair and replace lost cells have been investigated (Thuret et al., 2006; Mothe and Tator, 2012). Oligodendrocytes (OLs) are myelin-forming cells in the central nervous system (Bunge, 1968); their loss following SCI leads to demyelination of surviving axons (Crowe et al., 1997; Rabchevsky and Smith, 2001; Warden et al., 2001; Beattie et al., 2002), suggesting these cells or their precursors (OL progenitor cells, OPCs) as candidates for cell-based therapy. However, the poor survival of transplanted OLs and OPCs and inhibition of axonal regeneration are two limitations to their application (Lü et al., 2010).

Schwann cells (SCs) are peripheral nervous system-derived myelin-forming cells that produce growth factors and cell adhesion molecules (Acheson et al., 1991; Chiu et al., 1991; Oudega and Xu, 2006). Our previous study showed that co-transplantation of SCs and OPCs promoted the survival, proliferation and migration of transplanted OPCs *in vivo* and improved neurological recovery following SCI (Hu et al., 2013). A subsequent *in vitro* study confirmed that SCs modulated the proliferation and migration of OPCs by secreting platelet-derived growth factor (PDGF)-AA and fibroblast growth factor-2 (FGF)-2 (Chen et al., 2015); moreover, we showed that PDGF-AA stimulated the proliferation of OPCs and their differentiation into OLs at later stages (Hu et al., 2008). We therefore speculated that PDGF-AA administration would have similar effects as SCs in OPC transplantation for SCI repair. The present study tested this hypothesis by examining the effect of transplanted PDGF-AA-overexpressing OPCs on tissue repair and recovery of neurological function in a rat model of SCI.

## Materials and Methods

### Animals

A total of 145 adult female Sprague-Dawley rats were used in this study. Of these, 140 8-week-old rats (weighing 220–250 g) were subjected to contusive SCI and five pregnant rats were used for OPC isolation and culture. All animal protocols were in accordance with the Guide for the Care and Use of Laboratory Animals and the Guidelines and Policies for Rodent Survival Surgery approved by the Animal Care and Ethics Committees of Bengbu Medical College.

### Embryonic OPC Isolation and Culture

OPCs were isolated from the spinal cords of rat embryos according to a previously published protocol (Ma et al., 2009; Hu et al., 2013), with some modifications. Briefly, OPCs were immunopanned from spinal cords on embryonic day 14 using a monoclonal anti-A2B5 IgM antibody (1:1; gift from Dr. Scott R. Whittemore, University of Louisville, KY, USA) and plated in poly-D-lysine/fibronectin-coated 10 × 12-cm culture dishes with Dulbecco’s Modified Eagle’s Medium (DMEM)/Ham’s F12 (D/F12; Invitrogen, Carlsbad, CA, USA), 1× N2, 1× B27 supplement (Invitrogen), FGF-2 (20 ng/ml) and PDGF-AA (10 ng/ml). The medium was replaced every other day. After 5–7 days, cells were passaged and OPC purity was evaluated; the cultures were considered highly pure when >95% of cells expressed A2B5. Passage 2 cells were used for experiments.

### Preparation of Lentiviral Vectors Expressing PDGF-AA and Enhanced Green Fluorescent Protein (EGFP)

The lentiviral vectors pLenti6.3-enhanced green fluorescent protein (EGFP) and pLenti6.3- PDGF-AA-EGFP encoding rat EGFP and PDGF-AA cDNA, respectively, were generated by cloning into the pLenti6.3/v5 DEST backbone vector (Invitrogen). High-titer virus was obtained from human embryonic 293T cells seeded at 6 × 10^6^/10-cm plate in DMEM and cultured for 16 h. pLenti6.3-EGFP or pLenti6.3- PDGF-AA-EGFP along with the packaging plasmids pLP1, pLP2 and pLP/VSVG (Invitrogen) were co-transfected into 293T cells using Lipofectamine 2000 (Invitrogen) according to the manufacturer’s instructions. After 48 h, lentivirus was collected and concentrated from the medium by ultracentrifugation as described in our previous report (Hu et al., 2012). For lentiviral vector transduction, OPCs were cultured in growth medium for 24 h, and then exposed to pLenti6.3-EGFP or pLenti6.3- PDGF-AA-EGFP with a multiplicity of infection of 15; 24 h later, the medium was replaced with fresh medium and after another 24 h, transduction efficiency was estimated by EGFP expression under an epifluorescence microscope. After 5 days of culture, cells were passaged and fresh medium containing blasticidin (5 μg/ml) was added for selection of stably transduced cells. Lentivirus-infected OPCs were identified under proliferation and differentiation conditions by immunocytochemistry. OPCs transduced with pLenti6.3-EGFP or pLenti6.3- PDGF-AA-EGFP lentiviral vector are henceforth referred to as GFP-OPCs and PDGF-AA-OPCs, respectively.

### PDGF-AA-OPC Transplantation into Rats

Rats were subjected to contusive SCI using the weight-drop method (Gruner, 1992; Hu et al., 2013). Briefly, dorsal laminectomy at the T10 level was carried out on anesthetized adult female rats, and the dorsal surface of the spinal cord was subjected to a weight-drop impact of 10 g from a height of 12.5 mm. After SCI, muscles and skin were closed in layers, and rats were placed in a temperature- and humidity-controlled chamber. Rats received an analgesic agent buprenorphine (0.3 mg/kg) twice a day for 3 days to alleviate pain. Manual bladder emptying was performed three times daily until reflex bladder emptying was established. To prevent infections, animals were daily provided with chloramphenicol (50–75 mg/kg) via drinking water. Cell transplantation was performed on day 8 post-SCI. A total of 140 rats were randomly assigned to one of three groups: (1) control (OPC medium; *n* = 40); (2) GFP-OPC (2 × 10^5^; *n* = 50); and (3) PDGF-AA-OPC (2 × 10^5^; *n* = 50). Animals were re-anesthetized and the laminectomy site was re-exposed. A total of five injections were made into the spinal cord: one at the lesion center (2 μl of cell suspension at a concentration of 10^5^ cells/μl), two at 2 mm rostral to the center on either side, and two at 2 mm caudal to the center on either side at a depth of 1.2 and 0.6 mm laterally from the midline (0.5 μl/injection/side). Animals received daily subcutaneous injections of cyclosporine A (10 mg/kg; Sigma, St. Louis, MO, USA) starting 3 days prior to the transplantation until week 7 after transplantation, when the rats were sacrificed.

### Behavioral Assessment

Open-field locomotor testing was carried out by two trained investigators using the 21-point Basso, Bresnahan and Beattie (BBB) locomotor scale (Basso et al., 1995) once a week post-injury to assess hindlimb locomotor recovery including joint movement, stepping ability, coordination and trunk stability. Animals with a minimum BBB score of 12 at 6 weeks after cell transplantation were further analyzed and subjected to grid-walk analyses as described in our previous report (Hu et al., 2013).

### Tissue Preparation

At predetermined time points (1-, 2-, 4- and 7-week post-transplantation), rats were anesthetized with 60 mg/kg pentobarbital and transcardially perfused with 4% paraformaldehyde (PFA) in 0.01 M phosphate-buffered saline (PBS, pH 7.4). Spinal cord segments containing grafts were removed, post-fixed in the same fixative overnight at 4°C, and cryoprotected in 30% sucrose (Sigma) buffer for 5–7 days. A 2 cm-length of the spinal cord centered at the injection or injury site was dissected and embedded in HistoPrep (Fisher Scientific, Pittsburgh, PA, USA) on dry ice. After that the spinal cords were blocked, serial 20-μm-thick sections through the entire injury site were cut on a cryostat. At 1-, 2- and 4-week post-transplantation, all spinal cords from each group (*n* = 10/group) were cut as transverse sections; at 7 week post-transplantation, the spinal cords of 10 animals in each group were cut as transverse sections, five animals were cut as longitudinal sections and five animals were used for immunoelectron microscopy assay in cell-grafted groups, respectively. Sections were mounted on gelatin-coated slides (Fisher Scientific) and stored at −70°C.

### Histological Analysis

Two sets of transverse sections (each set containing serial sections spaced 500 μm apart) from rat spinal cord at 7 weeks post-SCI were stained with Luxol Fast Blue (LFB; Sigma) for white matter sparing analysis (*n* = 10/group) and cresyl violet-eosin (Sigma) for lesion volume assessment (*n* = 10/group), as previously described (Hu et al., 2013). White matter sparing was defined as tissue exhibiting normal myelin appearance and density; the lesion center was defined as the section containing the least amount of spared white matter. The areas of whole transverse sections and myelinated white matter at the lesion center were outlined and quantified in LFB-stained sections, and are expressed as a percentage of the total stained area. After cresyl violet-eosin staining, total and cross-sectional areas of the spinal cord and lesion boundary were measured using the Neurolucida System (MicroBrightField, Colchester, VT, USA) connected to a BX60 microscope (Olympus, Tokyo, Japan). The total volume of the lesion area (including areas of cavitation and degeneration) was calculated as the sum of the individual sub-volumes, which were determined by multiplying the cross-sectional area by the distance between sections (500 μm). The percent total volume of the injured area was calculated by dividing the total volume of the lesion area by that of the corresponding length of spinal cord.

### Immunohistochemistry

Frozen sections collected on slides were air-dried at room temperature for 10 min and washed with PBS for 10 min, then blocked with Tris-buffered saline containing 10% donkey serum and 0.3% Triton X-100 for 1 h at room temperature. Primary antibodies in the same blocking solution were applied overnight at 4°C. Mouse anti-activated caspase-3 antibody (1:1000; Sigma) was used to detect cell apoptosis; mouse anti-adenomatous polyposis coli (CC1, 1:100; Calbiochem, La Jolla, CA, USA), mouse anti-glial fibrillary acidic protein (GFAP, 1:200; Sigma), and mouse anti-neurofilament-H (SMI-31, 1:1000; Chemicon, Billerica, MA, USA) antibodies were used to detect OLs, astrocytes and axons, respectively. The slides were washed three times in PBS and incubated with fluorescein isothiocyanate- or rhodamine-conjugated goat anti-mouse IgG (all at 1:200; Jackson ImmunoResearch, West Grove, PA, USA) for 1 h at 37°C. Slides were washed three times with PBS and mounted with Gel/Mount containing Hoechst 33342 to counterstain nuclei. Images were acquired with a BX60 fluorescence microscope or Zeiss 510 laser confocal microscope (Oberkochen, Germany). Control samples were prepared by omitting the primary antibody.

### Bromodeoxyuridine (BrdU) Incorporation Assay

To assess OPC proliferation after transplantation, 3 days before the end of the 1-, 2- and 4-week post-transplantation time points, randomly selected rats (*n* = 10/group) were given 10 intraperitoneal injections of bromodeoxyuridine (BrdU; 50 mg/kg/injection; three times daily for 3 days and one injection on the last day) and were sacrificed 2 h after the last injection. The animals were perfused, their spinal cords were dissected, and the tissue was sectioned. One set of serial sections was randomly selected for the BrdU incorporation assay. Fixed sections were treated with 1 N HCl for 40 min at 37°C to denature the DNA, and a rabbit anti-BrdU antibody (1:100; Sigma) was applied overnight at 4°C, followed by rhodamine-conjugated donkey anti-rabbit IgG (1:200; Sigma) as a secondary antibody at room temperature for 2 h. BrdU+ cells were imaged under a BX60 microscope and at least five randomly selected fields in each section with a total of more than 500 GFP+ cells were counted. The percentage of BrdU+ GFP+ cells of the total number of GFP+ cells was determined.

### *In Vivo* Cell Migration Analysis

To analyze the migration ability of grafted cells, we checked all the longitude sections of all animals with cell transplantation under Olympus BX60 microscope. We measured the longest distal distance of the GFP^+^ cells from the injection sites which were 2 mm caudal or rostral to the epicenter and then calculated the average maximum migration distance of each group (*n* = 5).

### Immunoelectron Microscopy

Rats were transcardially perfused with 4% PFA and 0.15% glutaraldehyde in phosphate buffer (PB). Spinal cords were postfixed in 4% PFA in 0.1 M PB at 4°C, then embedded in 3% agarose A (biotechnology grade; Rose Scientific, Edmonton, AB, Canada) for vibratome sectioning. Free-floating sections (100 μm) of the spinal cord were processed for immunoperoxidase staining by washing three times in PBS and preincubating in 0.1% H_2_O_2_ in PBS for 15 min to quench the endogenous peroxidase activity. The sections were blocked in 5% milk, 1% bovine serum albumin, and 0.05% Triton X-100 in PBS for 3 h at room temperature and incubated in rabbit anti GFP antibody (1:100; Chemicon) overnight at 4°C. After three washes in PBS, sections were incubated in horseradish peroxidase-conjugated anti-rabbit secondary antibody (1:200) overnight at 4°C. After washing in PBS, the sections were postfixed in 0.1% glutaraldehyde for 10 min at room temperature and washed three times in 0.1 M PB. The slides were incubated in 0.04% diaminobenzidine (DAB) for 30 min, followed by a second incubation in 0.04% DAB with 0.005% H_2_O_2_ for 15 min. The sections were washed in PB for 15 min, treated with 1% osmium tetroxide in 0.1 M PB overnight, dehydrated in a graded series of ethanol solutions, and embedded in Araldite 502/Embed-812 medium (Electron Microscopy Sciences, Hatfield, PA, USA). Thin sections were cut on an ultramicrotome, counterstained with uranyl and lead citrate, and examined by transmission electron microscopy (Hitachi 7000; Hitachi, Tokyo, Japan). A negative control was prepared by omitting the anti-GFP antibody.

### Statistical Analysis

Data are presented as mean ± standard deviation. One-way analysis of variance (ANOVA) with Tukey’s Honestly Significant Difference or Fisher’s Least Significant Difference *post hoc* test was used to evaluate mean differences. BBB score was analyzed using repeated measure ANOVA, followed by Tukey’s pairwise comparison at each time point. Other data were analyzed using non-parametric Kruskal-Wallis ANOVA, followed by individual Mann-Whitney U tests. A *P* value <0.05 was considered statistically significant. Data were analyzed using SPSS v.14.0 software (SPSS Inc., Chicago, IL, USA).

## Results

### Transplantation of PDGF-AA-Overexpressing OPCs Promotes Functional Recovery after SCI

**Figure 1:**
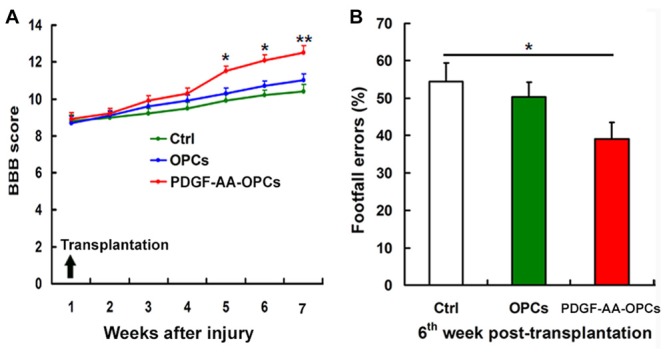
**Transplantation of platelet-derived growth factor (PDGF)-AA-overexpressing oligodendrocyte progenitor cells (OPCs) improves locomotor recovery after spinal cord injury (SCI). (A)** Rats that received PDGF-AA-overexpressing OPC transplantation showed greater improvement in locomotor Basso, Bresnahan and Beattie (BBB) score at 3 weeks as compared to controls (*n* = 15). **(B)** The PDGF-AA-overexpressing OPC transplantation group had fewer footfall errors in the grid-walk analysis than control animals 6 weeks post-transplantation. Data represent mean ± SD (*n* = 15). *P < 0.05, **P < 0.01.

One day after SCI, all injured rats were paraplegic with no observable hindlimb movement (BBB = 0, data not shown). We assessed the effect of transplanted PDGF-AA-overexpressing OPCs on functional recovery following SCI with the BBB test and grid-walking analyses. One week after injury (1 day before transplantation), the mean BBB score was 8.80 ± 0.51. In the first 3 weeks after transplantation, there was no difference among groups in terms of BBB score (Figure 1A). However, with continued recovery, rats transplanted with PDGF-AA-OPCs showed a significant improvement in BBB score as compared to the OPC only or control groups at 4–6 weeks post-transplantation (*P* < 0.05, *n* = 15; Figure 1A).

Rats were evaluated with the gridwalk test 6 weeks after transplantation. OPC transplantation alone did not improve grid walking performance as compared to the control group (Figure 1B). However, PDGF-AA-OPC transplantation decreased the number of footfall errors as compared to the OPC only or control groups (*P* < 0.05, *n* = 15; Figure 1B).

### Transplantation of PDGF-AA-Overexpressing OPCs Increases Tissue Sparing in Injured Spinal Cord

**Figure 2:**
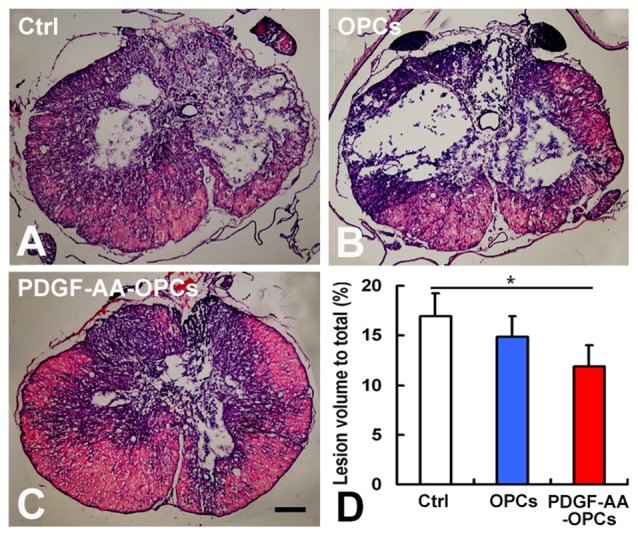
**Tissue sparing in the spinal cord following SCI. (A–C)** Representative images of sections stained with cresyl violet-eosin showing the extent of tissue sparing at the lesion center 7 weeks post-transplantation. **(D)** Quantitative analysis of relative lesion volume (*n* = 10). *P < 0.05. Scale bar: 200 μm.

We examined the gross morphology of the injured spinal cord 7 weeks post-transplantation to determine whether transplantation of PDGF-AA-overexpressing OPCs can increase tissue sparing. We found that lesion volume was markedly decreased upon transplantation of PDGF-AA-overexpressing OPCs relative to control animals (*P* < 0.05, *n* = 10; Figures 2A–D).

### PDGF-AA Overexpression Promotes the Survival of Transplanted OPCs in Injured Spinal Cord

**Figure 3:**
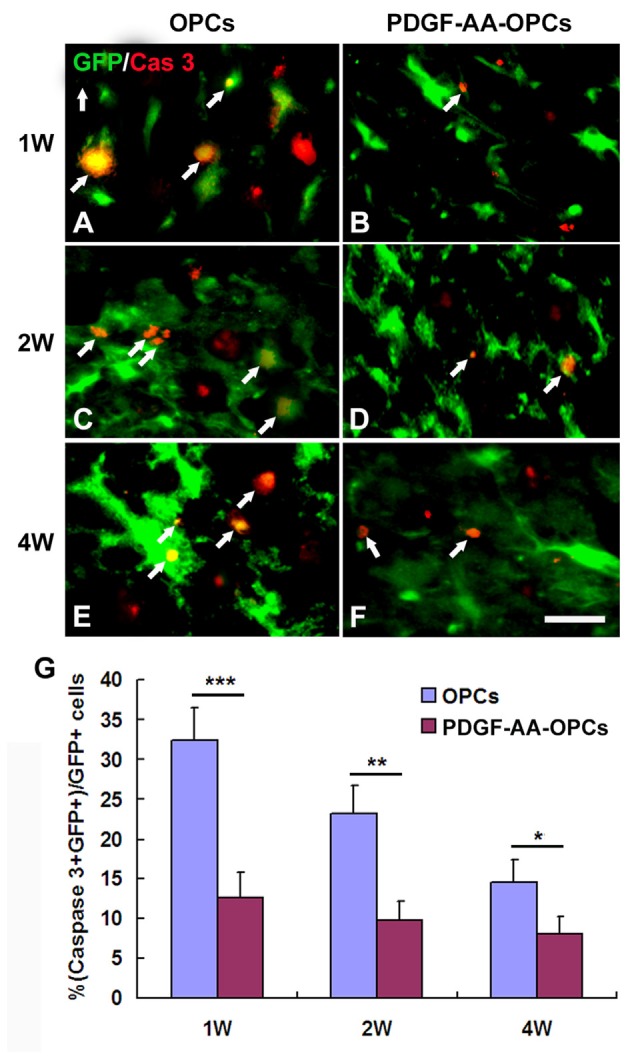
**Survival of OPCs in the injured spinal cord after transplantation, as determined by activated caspase-3 staining. (A–F)** Representative photomicrographs showing caspase-3+ cells (red) co-localized with green fluorescent protein (GFP+) OPCs (green) in the spinal cord of rats that received transplantation of OPCs with or without PDGF-AA overexpression at 1, 2, or 4 weeks. **(G)** Quantitative analysis of activated caspase-3+ cells. There were fewer caspase-3+ GFP+ cells in the PDGF-AA-overexpressing OPC transplantation group than in the OPC transplantation groups. Data represent mean ± SD (*n* = 10). *P < 0.05, **P < 0.01, ***P < 0.001. Scale bar = 25 μm.

Detection of activated caspase-3 at 1, 2 and 4 weeks post-transplantation revealed that PDGF-AA overexpression promoted the survival of transplanted OPCs in the injured spinal cord as compared to the OPC group (*P* < 0.05; Figures 3A–G). There were fewer caspase-3+ GFP+ cells among total GFP+ cells in the PDGF-AA-overexpressing OPC transplantation group as compared to the OPC group at 1 week (12.63% ± 3.20% vs. 32.35% ± 4.13%, *P* < 0.001, *n* = 10), 2 weeks (9.78% ± 2.45% vs. 23.20% ± 3.54%, *P* < 0.01, *n* = 10), and 4 weeks (8.13% ± 2.15% vs. 14.52% ± 2.87%, *P* < 0.05, *n* = 10; Figure 3G).

### Overexpression of PDGF-AA Promotes Proliferation of Transplanted OPCs in Injured Spinal Cord

**Figure 4:**
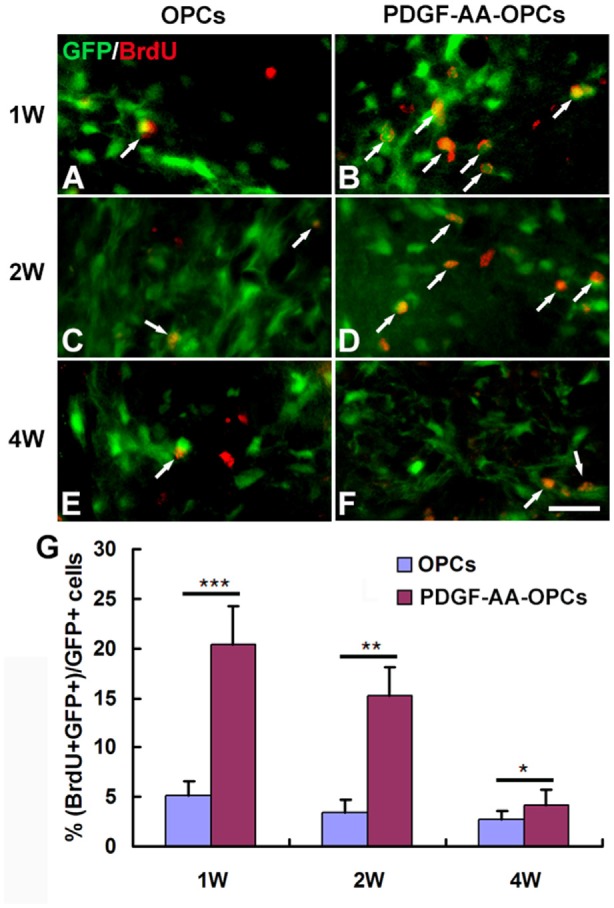
**Proliferation of transplanted OPCs in the injured spinal cord, as determined by bromodeoxyuridine (BrdU) incorporation. (A–F)** Representative photomicrographs showing BrdU+ cells (red) co-localized with GFP+ OPCs (green) in the spinal cord of rats transplanted with OPCs with or without PDGF-AA overexpression at 1, 2 and 4 weeks. **(G)** Quantitative analysis of OPC proliferation. The number of BrdU+ OPCs was higher in rats that received transplantation of OPCs overexpressing PDGF-AA as compared to those that did not express PDGF-AA. Data represent mean ± SD (*n* = 10). *P < 0.05, **P < 0.01, ***P < 0.001. Scale bar = 25 μm.

The BrdU incorporation assay was used to assess the proliferation of transplanted OPCs. There were more BrdU+ GFP+ cells among total GFP+ cells in the PDGF-AA-overexpressing OPC transplantation group as compared to the OPC group at 1 week (20.37% ± 3.85% vs. 5.20% ± 1.34%, *P* < 0.001, *n* = 10), 2 weeks (15.25% ± 2.82% vs. 3.50% ± 1.24%, *P* < 0.01, *n* = 10), and 4 weeks (4.12% ± 1.62% vs. 2.70% ± 0.90%, *P* < 0.05, *n* = 10; Figure 4).

### Overexpression of PDGF-AA Promotes Migration of Transplanted OPCs in Injured Spinal Cord

**Figure 5:**
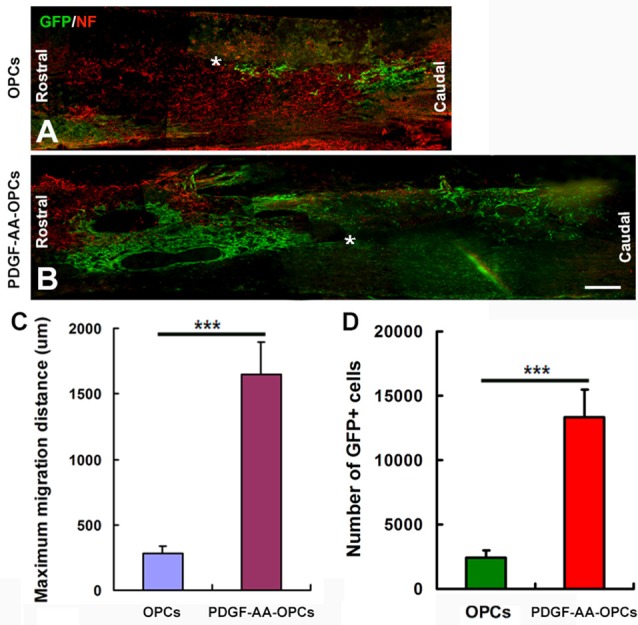
**Migration of transplanted OPCs in the spinal cord after transplantation. (A–D)** Representative longitudinal sections showing the migration of transplanted OPCs over longer distances from the injury center (*) when overexpressing PDGF-AA as compared to those without PDGF-AA overexpression at 7 weeks. Data represent mean ± SD (*n* = 5). ***P < 0.001. Scale bar = 500 μm.

The migration of OPCs in the injured spinal cord was examined 7 weeks after transplantation (Figures 5A–D). Numerous GFP+ OPCs had migrated away from the injury center in rats that received transplantation of PDGF-AA-overexpressing OPCs (Figure 5B). These results indicate that PDGF-AA overexpression stimulated migration of transplanted OPCs as compared to those that did not express PDGF-AA (Figures 5A–C). Moreover, the number of transplanted GFP+ OPCs was higher in the PDGF-AA-overexpressing OPC transplantation as compared to the OPC group (Figures 5A,B,D).

### Overexpression of PDGF-AA Stimulates Differentiation of Transplanted OPCs into OLs

**Figure 6:**
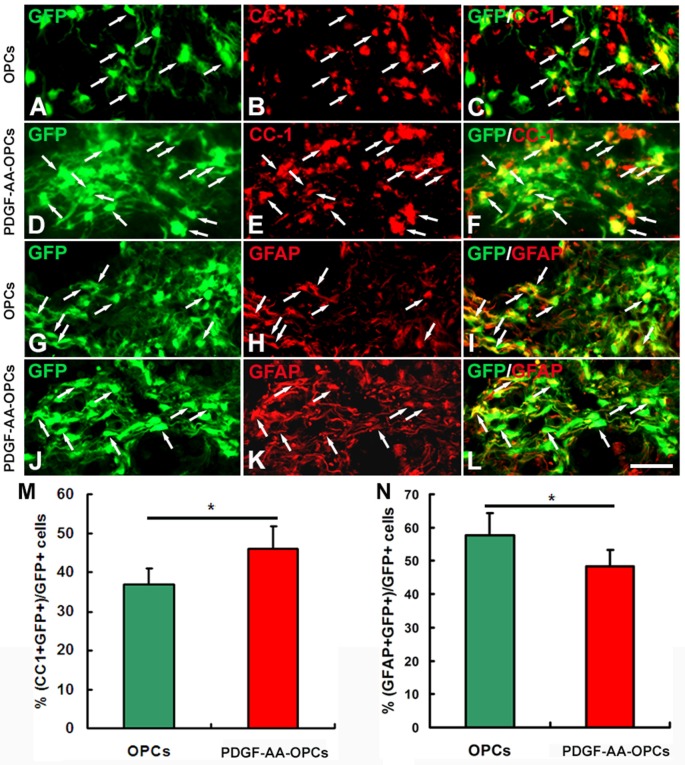
**Differentiation of transplanted OPCs in the injured spinal cord. (A–K)** Representative images showing differentiation of transplanted GFP+ OPCs into CC1+ oligodendrocytes (OLs) in the spinal cord 7 weeks post-transplantation in the non-PDGF-AA-overexpressing OPC transplantation group **(A–C)** and PDGF-AA-overexpressing OPC transplantation group **(D–F)**. **(G–L)** Representative images showing differentiation of transplanted GFP+ OPCs into astrocytes in the spinal cord 7 weeks post-transplantation in the non-PDGF-AA-overexpressing OPC transplantation group **(G–I)** and PDGF-AA-overexpressing OPC transplantation group **(J,K)**. **(M,N)** Quantitative analysis of CC1+ OLs **(G)** and GFAP+ astrocytes. Significant differences in the percentage of CC1+ OLs **(M)** and GFAP+ astrocytes **(N)** were observed between the two transplantation groups. Data represent mean ± SD (*n* = 10). Scale bar = 25 μm. *P < 0.05.

To assess the effect of PDGF-AA overexpression on the differentiation of transplanted OPCs, we compared the numbers of transplanted GFP-OPCs and PDGF-AA-overexpressing OPCs that differentiated into CC1+ OLs and GFAP+ astrocytes 7 weeks after transplantation (Figure 6). The percentage of CC1+ OLs was higher in the PDGF-AA-OPC group (46.20% ± 5.50%, *P* < 0.05, *n* = 10), than in the OPC group (36.81% ± 4.20%, *P* < 0.05, *n* = 10; Figures 6A–F,M). Conversely, the percentage of GFAP+ astrocytes was lower in the PDGF-AA-overexpressing OPCs group (48.30% ± 4.92%, *P* < 0.05, *n* = 10) as compared to the OPC group (57.81% ± 6.50%, *P* < 0.05, *n* = 10; Figures 6G–L,N). These findings indicate that PDGF-AA overexpression induces the differentiation of transplanted OPCs into OLs.

### Transplantation of PDGF-AA-Overexpressing OPCs Increases Myelination in Injured Spinal Cord

**Figure 7:**
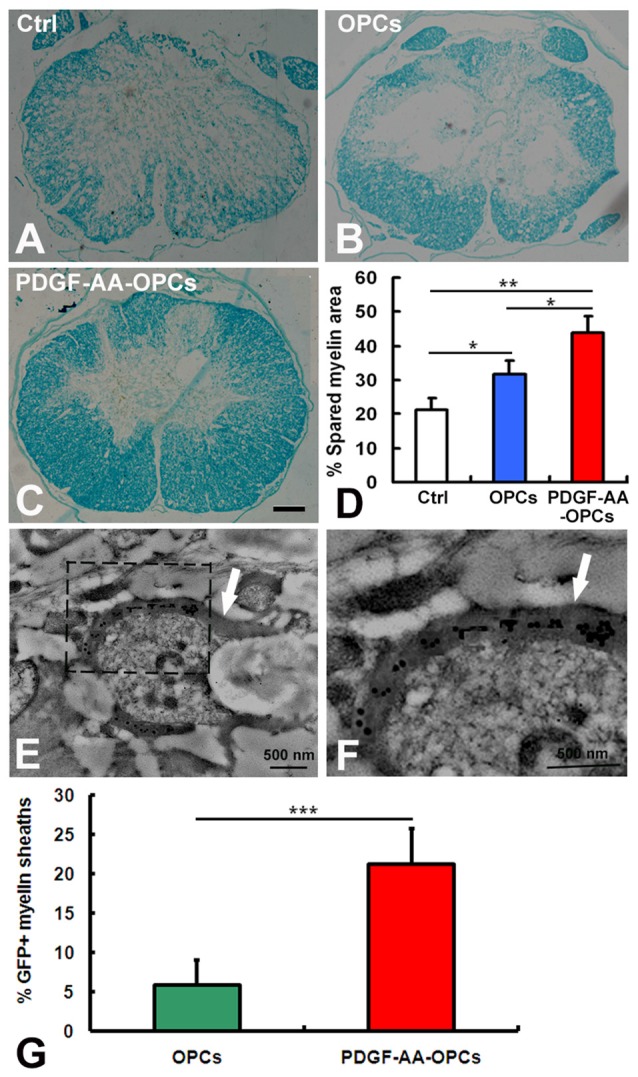
**Detection of myelination and remyelination by luxol fast blue (LFB) staining and immunoelectron microscopy in rats with SCI 7 weeks after OPC transplantation. (A–C)** Representative images of stained sections showing the extent of myelination at the injury center 7 weeks after transplantation. **(D)** Quantitative analysis of myelinated areas (*n* = 10, *P < 0.05). **(E,F)** Ultrastructural analysis of an OPC-derived mature oligodendrocyte near newly formed central myelin sheaths (arrows). GFP immunoreactivity is clearly seen within the myelin sheaths, confirming their origin from a transplanted OPC. **(G)** Quantitative analysis of GFP^+^ myelin sheaths showed a significant difference between the non-PDGF-AA-overexpressing OPC transplantation group and the PDGF-AA-overexpressing OPC transplantation group. **P < 0.01; ***P < 0.001. Scale bar: 200 μm in **(A–C)**.

To investigate whether transplantation of PDGF-AA-overexpressing OPCs better preserve existing myelin and/or promote remyelination, LFB staining was performed to assess the extent of residual or newly formed myelin at the injury center 7 weeks after transplantation. Although transplantation of OPCs increased the area of LFB staining at the injury center relative to the control group (*P* < 0.05, *n* = 10), the area was greater in rats transplanted with OPCs overexpressing PDGF-AA as compared to those that did not expression PDGF-AA (*P* < 0.01, *n* = 10; Figures 7A–D). We further assessed the ability of OLs newly generated from transplanted OPCs to form myelin on existing or regenerated axons by immunoelectron microscopy 7 weeks post-transplantation. Our results revealed GFP immunoreactivity in newly formed myelin sheaths generated by OLs derived from transplanted OPCs (Figures 7E,F). There was a higher percentage of the GFP^+^ myelin sheaths in the PDGF-AA-overexpressing OPC transplantation group as compared to the OPC transplantation group (Figure 7G).

## Discussion

We previously showed that co-transplantation of SCs promoted the survival, proliferation and migration of transplanted OPCs and enhanced functional recovery after SCI (Hu et al., 2013). SCs modulate OPC proliferation and migration via secretion of PDGF-AA and FGF-2 (Chen et al., 2015). In the present study, we investigated whether transplantation of OPCs overexpressing PDGF-AA could improve functional recovery after SCI. In the first 3 weeks after transplantation, there was no difference in BBB score among groups. However, at 4–6 weeks post-transplantation, there was a significant improvement in the BBB score in rats that received PDGF-AA-overexpressing OPCs as compared to OPCs that did not overexpress PDGF-AA. We also found that rats in the PDGF-AA-overexpressing OPC transplantation group showed greater improvement in the gridwalk test, another functional measure, at week 6 as compared to the other groups. These behavioral findings are consistent with our previous results of OPC/SC co-transplantation, suggesting that the effect of PDGF-AA overexpression in OPCs is similar to SC co-transplantation (Hu et al., 2013) and is a potential alternative strategy for SCI treatment.

We next evaluated whether transplantation of PDGF-AA-overexpressing OPCs can promote tissue repair in the injured spinal cord. We observed a significant reduction in spinal cord lesion volume in rats that received with PDGF-AA-overexpressing OPCs as compared to non-PDGF-AA-overexpressing OPCs or control rats, indicating that PDGF-AA overexpression in OPCs enhances tissue repair following SCI and explaining the functional improvement observed in these animals.

To investigate the mechanism by which PDGF-AA overexpression promotes functional recovery and tissue repair following SCI, we examined the growth of transplanted OPCs overexpressing PDGF-AA. Activated caspase-3 labeling and BrdU incorporation revealed that PDGF-AA overexpression protected OPCs from apoptosis and promoted their survival and proliferation after transplantation. Moreover, these cells migrated longer distances than non-PDGF-AA-overexpressing OPCs and showed increased OL differentiation at 7 weeks. These results suggest that PDGF-AA overexpression in OPCs induces the differentiation of OPCs into OLs instead of astrocytes at a later stage after transplantation. This result contradicts our previous observation that SCs did not affect OL differentiation of co-transplanted OPCs at the site of injury (Hu et al., 2013), but is in agreement with our earlier study that PDGF-AA induced proliferation and possibly differentiation at later stages of OL development *in vitro* (Hu et al., 2008). Taken together, our results suggest that PDGF-AA overexpression promotes the survival, proliferation and migration of OPCs at an earlier stage and enhances OL differentiation at a later stage after transplantation.

Functional recovery following SCI depends on preservation of existing myelin and remyelination (Keirstead et al., 2005; Xu and Onifer, 2009). In the present study, there was more residual myelin at the injury center in rats that received PDGF-AA-overexpressing OPC transplantation as compared to non-PDGF-AA-overexpressing OPCs. Immunoelectron microscopy analysis revealed GFP immunoreactivity in newly formed myelin sheath produced by transplanted OPC-derived OLs at 7 weeks, indicating that PDGF-AA overexpression in OPCs preserves residual myelin and promotes remyelination of demyelinated axons when transplanted into the injured spinal cord.

As previously mentioned, SCs promote OPC proliferation and migration through secreting PDGF-AA and FGF-2 (Chen et al., 2015). In the present study, we only explored the effect of PDGF-AA-overexpressing OPCs on SCI. Whether FGF-2-overexpressing OPCs also have a role in SCI repair remains unknown. Moreover, it must be noted that there is still the possibility that PDGF-AA which is secreted transplanted PDGF-AA-overexpressing OPCs could affect endogenous OPCs and thus promote SCI repair. These questions will be explored in our future study.

In conclusion, our results demonstrate that PDGF-AA overexpression in OPCs promoted the survival, proliferation, migration and OL differentiation of OPCs *in vivo*, which increased myelination and tissue repair in the injured spinal cord, leading to the recovery of neurological function. These results suggest that transplanting PDGF-AA-overexpressing OPCs is a potentially effective strategy for SCI treatment.

## Author Contributions

J-GH and H-ZL designed and supervised the project. Z-FY, YiW, Y-HL, YaW, LS, JX and QQ performed research. A-YZ, RW and Z-QJ analyzed data and Z-FY and YiW wrote the article.

## Conflict of Interest Statement

The authors declare that the research was conducted in the absence of any commercial or financial relationships that could be construed as a potential conflict of interest.
